# Editorial: Innovative approaches for assessing and improving perioperative neurocognitive disorders

**DOI:** 10.3389/fnagi.2022.1098250

**Published:** 2022-12-09

**Authors:** Susanne Koch, Jose I. Egaña, Antonello Penna, Beverley A. Orser, Patrick L. Purdon, Rodrigo Gutiérrez

**Affiliations:** ^1^Department of Anaesthesiology and Operative Intensive Care Medicine, Charité Universitätsmedizin Berlin, Berlin, Germany; ^2^Department of Anesthesia and Perioperative Medicine, Faculty of Medicine, University of Chile, Santiago, Chile; ^3^Centro de Investigación Clínica Avanzada, Hospital Clínico de la Universidad de Chile, Santiago, Chile; ^4^Department of Physiology, University of Toronto, Toronto, ON, Canada; ^5^Sunnybrook Health Sciences Centre, Toronto, ON, Canada; ^6^Department of Anesthesiology and Pain Medicine, Temerty Faculty of Medicine, University of Toronto, Toronto, ON, Canada; ^7^Perioperative Brain Health Centre, Sunnybrook Health Sciences Centre, Toronto, ON, Canada; ^8^Department of Anesthesia, Critical Care and Pain Medicine, Massachusetts General Hospital, Boston, MA, United States; ^9^Harvard Medical School, Boston, MA, United States

**Keywords:** anesthesia, delirium, electroencephalogram, cognition, elderly

The adverse cerebral effects of general anesthetic drugs and surgery in older people have been recognized for more than 50 years (Bedford, 1955). Deficits related to cognition have garnered attention in the fields of anesthesiology and critical care medicine in the past decade (Evered and Silbert, 2018). This Research Topic resulted from an effort to gather new evidence about postoperative neurocognitive disorders (PNDs) (Evered et al., 2018). The articles included in this Research Topic cover a broad range of themes, including prediction of PNDs, intraoperative neurophysiological characterization of the aging brain, and clinical description of the problem (Figure 1).

**Figure 1:**
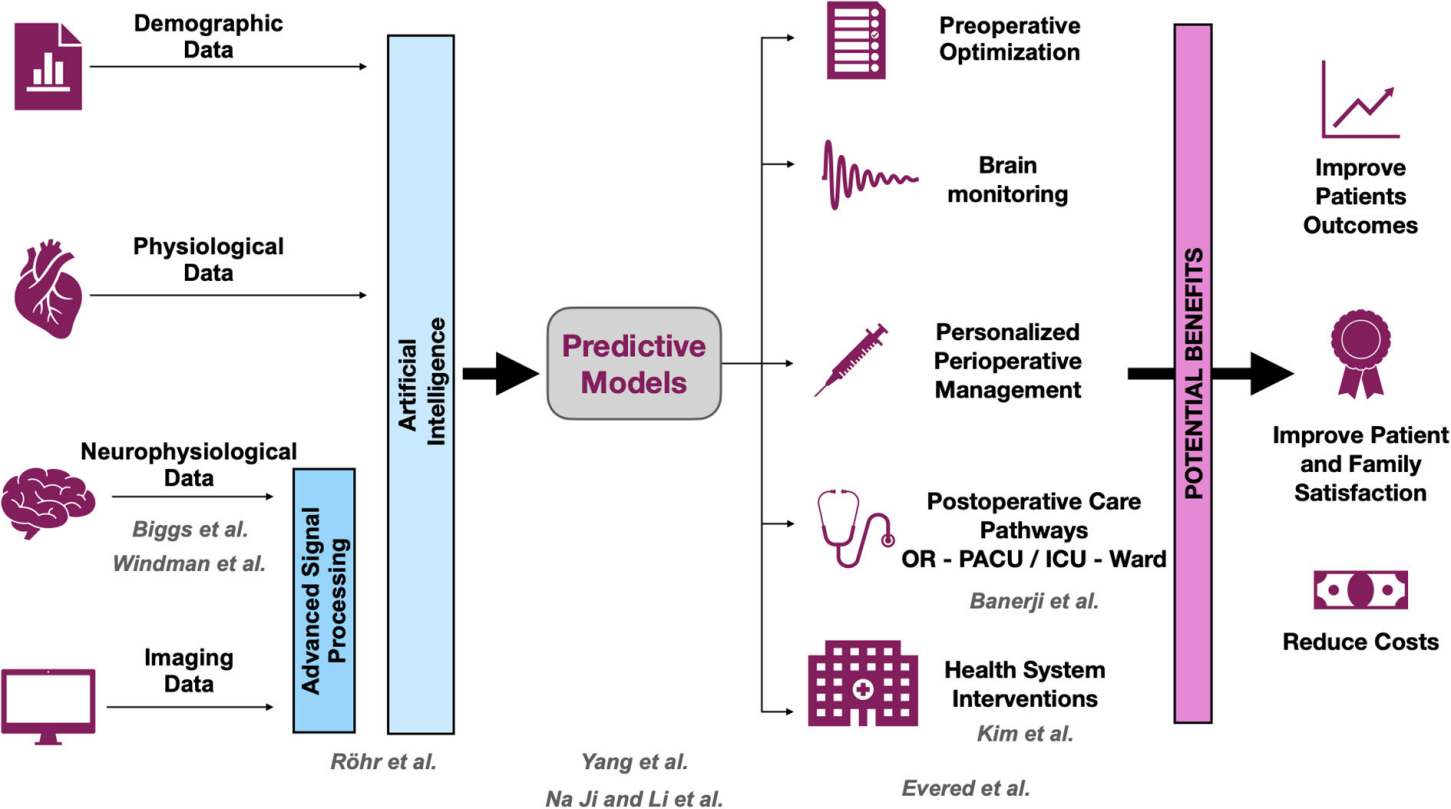
Opportunities and benefits to improve postoperative neurocognitive disorders (PNDs). The scheme shows how research could help to improve the outcomes associated with PND. The development of predictive models is crucial for planning strategies to mitigate PND. All contributions that are part of this Research Topic cover at least one of the levels, and they are displayed in gray. OR, operating room; PACU, post-anesthesia care unit; ICU, intensive care unit.

Better prediction models are needed to identify patients at risk of various types of PND, including postoperative delirium (POD). This information will help clinicians to adjust their management and to direct particular efforts toward patients needing greater assistance. This Research Topic includes several articles concerning PND prediction. Using clinical data, Yang et al. developed a predictive model for POD that can be easily implemented by clinicians. They found that dementia, low serum albumin levels, and chronic obstructive pulmonary disease were key predictors of POD. Interestingly, these authors also presented a visual tool to support the application of the model, based on a nomogram. Röhr et al. developed a different prediction model for POD based on a machine learning approach. They used clinical data and intraoperative electroencephalographic (EEG) signals to develop their prediction model. Their results highlight the importance of using non-invasive technology, such as EEG, to improve the performance of such models. Importantly, these authors cross-validated their results, using their model in a different sample. Finally, Ji and Li present a detailed bibliometric analysis of published studies that examined predictions of PND after cardiac surgery. Two important messages can be taken from their study. First, the number of publications on this topic has continued to increase over the past 20 years, providing evidence of general interest. Second, according to the literature, one of the most active research in terms of PND prediction is associated with the identification of inflammatory biomarkers.

In two other studies reported in this collection, researchers analyzed intraoperative EEG signals with the aim of understanding, at least in part, the neurophysiology underlying the aging brain and PND. Windman et al. demonstrated that a stronger shift in the direct-current (DC) EEG during induction of anesthesia is associated with increased risk of POD. Patients affected by PND showed a large negative DC deflection relative to control patients. Given that the same deflection is associated with changes in end tidal CO_2_, the authors suggest a potential link between delirium risk, cerebral metabolism, and changes in the EEG signal. In a separate study, Biggs et al. evaluated the effect of age on various metrics derived from the intraoperative EEG. These authors found that permutation entropy seems to remain unchanged with age, and therefore could be a promising candidate to serve as an age-independent measure of the unconscious state.

Banerji et al. provide a detailed description of the cognitive domain affected in patients experiencing delirium in the post-anesthesia care unit. Interestingly, specific cognitive domains were differentially affected across their study sample, attention being affected to the greatests extent. These results offer important insight into the neuropathologic process involved in POD, as well as the process of cognitive recovery after anesthesia. The impact of prohibiting patient visitors during the recent COVID-19 pandemic was investigated by Kim et al. Although policies restricting ICU visits during the pandemic seemed not to affect the incidence of delirium, the restrictions were associated with changes in delirium subtype and level of anxiety. The results of this study emphasize the potential impact of hospital-level decisions on the development of neurocognitive disorders.

Finally, to complete the Research Topic, Evered et al. present a comprehensive review covering recent key developments in the identification of PND and its management throughout the perioperative period. They also describe current hypotheses related to the development of PND and present a comprehensive brain-enhanced recovery pathway designed to prevent and mitigate PND, underlining the importance of the multidisciplinary teams involved in the perioperative care.

A synergetic relationship between fundamental preclinical neuroscience and clinical perioperative medicine has been key to gaining a better understanding of PND and developing new treatments. Anesthesiologists continue to play a key role in promoting measures to improve brain health in the perioperative period. A controversial, yet potentially modifiable factor associated with PND is the use of general anesthetic drugs. Safavynia et al. note in their review, that the choice of anesthetic drug and techniques (e.g., regional vs. general anesthesia) is not an independent risk factor for the development of impaired cognition after surgery. However, numerous studies of laboratory animals have consistently shown that anesthetic drugs cause deficits in cognition that persist after the drugs have been eliminated from the body, a finding recently corroborated by a scoping review (Guo et al., 2022). The potential benefits of reducing anesthetic exposure appear to depend on the context, reflecting the multifactorial nature of PND. For patients with hip fracture, regional anesthesia did not reduce delirium incidence relative to general anesthesia (Neuman et al., 2021). In contrast, EEG-based titration to light anesthesia significantly reduced delirium incidence relative to deep general anesthesia (Evered et al., 2021). Thus, the contribution of anesthetic drugs remains to be elucidated, particular for patients undergoing long surgical procedures and those with preexisting cognitive deficits. As several studies published in this Research Topic point out, EEG seems to be a valuable tool not only for predicting and assessing the development of PND, but also for understanding the fundamental mechanisms involved in brain aging. The use of the EEG to prevent the development of PND is still matter of considerable debate; however, most of the available evidence indicates that it is a useful tool (Chan et al., 2013; Radtke et al., 2013; Garcia, 2020; Evered et al., 2021).

We encourage all physicians and allied health care workers who contribute to perioperative medicine, especially those caring for geriatric patients, to continue their search for knowledge about advances related to PND. Overall, the field is expanding rapidly. Readers are also encouraged to access the Perioperative Brain Health Initiative website of the American Society of Anesthesiologists to obtain up-to-date information ASA. As proposed in the schematic shown in Figure 1, efforts should be invested in the prediction and early detection of patients at risk for PND, as well as in the development of new therapeutic approaches for managing patients who experience PND. The downstream benefits of early detection and therapy would not only improve patients' outcomes and satisfaction but also improve the efficiency of health systems and reduce health care costs.

## Author contributions

SK, BO, PP, and RG drafted the manuscript. JE and AP reviewed it. All authors contributed to the article and approved the submitted version.
